# A General Method for Site Specific Fluorescent Labeling of Recombinant Chemokines

**DOI:** 10.1371/journal.pone.0081454

**Published:** 2014-01-28

**Authors:** Tetsuya Kawamura, Bryan Stephens, Ling Qin, Xin Yin, Michael R. Dores, Thomas H. Smith, Neil Grimsey, Ruben Abagyan, JoAnn Trejo, Irina Kufareva, Mark M. Fuster, Catherina L. Salanga, Tracy M. Handel

**Affiliations:** 1 Skaggs School of Pharmacy and Pharmaceutical Sciences, University of California San Diego, La Jolla, California, United States of America; 2 Biomedical Sciences Graduate Program, School of Medicine, University of California San Diego, La Jolla, California, United States of America; 3 Marine Drug Research Institute, Huaihai Institute of Technology, Lianyungang, China; 4 Veterans Affairs San Diego Healthcare System, San Diego, California, United States of America; 5 Department of Medicine, Division of Pulmonary and Critical Care, University of California San Diego, La Jolla, California, United States of America; 6 Department of Pharmacology, University of California San Diego, La Jolla, California, United States of America; University of Edinburgh, United Kingdom

## Abstract

Chemokines control cell migration in many contexts including development, homeostasis, immune surveillance and inflammation. They are also involved in a wide range of pathological conditions ranging from inflammatory diseases and cancer, to HIV. Chemokines function by interacting with two types of receptors: G protein-coupled receptors on the responding cells, which transduce signaling pathways associated with cell migration and activation, and glycosaminoglycans on cell surfaces and the extracellular matrix which organize and present some chemokines on immobilized surface gradients. To probe these interactions, imaging methods and fluorescence-based assays are becoming increasingly desired. Herein, a method for site-specific fluorescence labeling of recombinant chemokines is described. It capitalizes on previously reported 11–12 amino acid tags and phosphopantetheinyl transferase enzymes to install a fluorophore of choice onto a specific serine within the tag through a coenzyme A-fluorophore conjugate. The generality of the method is suggested by our success in labeling several chemokines (CXCL12, CCL2, CCL21 and mutants thereof) and visualizing them bound to chemokine receptors and glycosaminoglycans. CXCL12 and CCL2 showed the expected co-localization on the surface of cells with their respective receptors CXCR4 and CCR2 at 4°C, and co-internalization with their receptors at 37°C. By contrast, CCL21 showed the presence of large discrete puncta that were dependent on the presence of both CCR7 and glycosaminoglycans as co-receptors. These data demonstrate the utility of this labeling approach for the detection of chemokine interactions with GAGs and receptors, which can vary in a chemokine-specific manner as shown here. For some applications, the small size of the fluorescent adduct may prove advantageous compared to other methods (e.g. antibody labeling, GFP fusion) by minimally perturbing native interactions. Other advantages of the method are the ease of bacterial expression, the versatility of labeling with any maleimide-fluorophore conjugate of interest, and the covalent nature of the fluorescent adduct.

## Introduction

Chemokine-mediated cell migration is a complex process involving many dynamic steps including transport of chemokines across cells, presentation of chemokines on cell surface glycosaminoglycans (GAGs) and extracellular matrix components, binding of chemokines to their G protein-coupled receptors (GPCRs), and scavenging and transport of chemokines by atypical chemokine receptors [1]–[4]. Binding to receptors typically involves monomeric forms of chemokines. However homo- and hetero- oligomerization has also been shown to play important roles in the overall regulation and function of the chemokine system [5]–[9]. In order to understand the complex interactions and dynamic mechanisms involved in chemokine biology and to track their spatial and temporal dependence, fluorescence and bioluminescent imaging methods have become important tools. In this study we focused on the development and application of exogenously added fluorescent chemokines, and to this end a number of studies have been previously reported: the fluorescent protein eGFP, the bioluminescent protein luciferase, and streptavidin coated fluorescent quantum dots have been fused or conjugated to the C-terminus of CXCL12 in investigations of the scavenging function of the chemokine receptor CXCR7 [10]–[12]. Similarly, CCL2-mCherry has been used to monitor the scavenging function of CCR2 during the migration of monocytes [13]. Fluorescent chemokines have also been used to detect binding interactions with receptors [14].

While these and other reports establish the utility of using fluorescent chemokines to study receptor interactions, many of the above examples involve fusions with large fluorescent/bioluminescent proteins that can interfere with interactions, and often require expression in eukaryotic cells which is time-consuming compared to bacterial expression systems. Additionally, some genetically encoded GFP-like fusions tend to oligomerize, which may be undesirable [15]. Detection with fluorescent anti-chemokine antibodies, while a method of choice for detecting endogenous chemokine, can be suboptimal if the antibody blocks interactions with other chemokines, GAGs or receptors, or does not recognize chemokines when they are complexed to other macromolecules.

To solve some of these problems, covalent labeling of chemokines with small organic dyes has been pursued [14]. Non-specific covalent labeling of chemokines through amine-coupling chemistry is the simplest method and is commonly used for antibodies and many other proteins. However, given that the N-termini of chemokines are critical for signaling [16] and that lysine residues are frequently involved in chemokine interactions with GAGs and chemokine receptors [17], labeling in this manner is likely to alter the function of the ligand. Accordingly, site-specific labeling methods are preferred. Along these lines, we recently introduced a non-native cysteine residue at the C-termini of chemokines CCL14/HCC-1(amino acids 9-74) and CCL7/MCP-3, and labeled them with maleimide conjugated fluorophores which are widely available with a broad array of emission wavelengths [18]. As expected, this strategy resulted in the production of fluorescent chemokines that were fully functional because modification of the C-termini, including attachment of fusion proteins, generally has little effect on receptor interactions. The disadvantage of this approach is that one must ensure that the extra cysteine does not interfere with correct folding and disulfide bond formation of the other native cysteine residues (all chemokines contain one-three disulfides, the majority have two). The method worked well for CCL7 and CCL14 because these chemokines are expressed in soluble form with no need for refolding to promote formation of the two structural disulfides, and they are well behaved in solution with little tendency to oligomerize. However, when applied to other chemokines, we encountered difficulties with aggregation and low yield due to the formation of disulfide-linked dimers or scrambled disulfides. Synthetic approaches for making site specifically labeled chemokines have been described and can limit inappropriate disulfide formation by the use of different protecting groups on the native and non-native cysteines [14]. While such chemokines are available commercially, they are quite expensive.

As an alternative approach we investigated the use of recently described genetically encoded peptide tags that can be labeled with phosphopantetheinyl transferase enzymes (PPTases) [19], [20]. The initial 11 amino acid tag was derived from *B. subtillis* and then subject to phage display to identify modified sequences that can be orthogonally labeled based on differential specificities of *B. subtillis* Sfp and *E. coli* AcpS PPTases. We focused primarily on the S6 tag, which is a good substrate for Sfp. In this report we describe the preparation of chemokine-fluorescent conjugates, demonstrate that they are functional, and illustrate their use in microscopy and flow cytometry-based approaches for interrogating interactions with receptors and GAGs.

## Materials and Methods

### Materials

All chemicals were purchased from Sigma-Aldrich, unless otherwise specified.

### Protein expression, purification and fluorescent labeling

For all chemokines used in this study, the DNA sequence coding for the S6 tag, GGCGATAGCCTGAGCTGGCTGCTGCGCCTGCTGAACTAA, (translation: GDSLSWLLRLLN-stop) was inserted immediately after the codon for the C-terminal residue. Chemokines were expressed and purified as described previously [18], [21]. The Sfp enzyme was a gift from Professor Jun Yin (University of Chicago) and was expressed and purified as described previously [22]. All fluorescent probes used in this study (Table 1) were purchased from Invitrogen, with the exception of Cy3B (GE Healthcare) and NPM (Sigma). The coenzyme A (CoA)-fluorophore conjugates were prepared as described previously [22], and lyophilized following purification by C18 reversed-phase HPLC. Prior to the labeling reaction, the stock concentration of the dye conjugate was determined by the maximum UV/VIS absorbance, using the extinction coefficients published by the dye manufacturers. Protein concentration was determined by the absorbance at 280 nm. The labeling reaction was initiated by mixing the S6-tagged chemokine, the CoA-fluorophore conjugate, and the Sfp enzyme at a final concentration of 10 µM, 10 µM, and 2 µM, respectively, in 50 mM HEPES, 10 mM MgCl_2_, pH 7.2. The reaction was incubated at room temperature in the dark for 30 min, and the labeled protein was lyophilized following purification by C18 reversed-phase HPLC. The molecular weights of the labeled products were validated by ESI mass spectrometry.

**Table 1:** Mass spectrometry validation of fluorescently labeled chemokines.

Fluorophore	Chemokine	ΔMW observed^a^	ΔMW expected^b^
Alexa Fluor 647	CXCL12	1323	NA
	LGG-CXCL12^c^	1324	NA
	CXCL12 K24S/H25S/K27S	1323	NA
	CCL2	1323	NA
	CCL7	1324	NA
	CCL21	1324	NA
	vMIP-II	1324	NA
Alexa Fluor 488	CXCL12	1040	1038
	CXCL12 K24S/H25S/K27S	1041	1038
	CXCL12 H25R	1040	1038
	CXCL12 P2G	1040	1038
Cy3B	CXCL12	1024	NA
	LGG-CXCL12^c^	1024	NA
NPM	CXCL12	639	638
	CCL2	640	638
CPM	CXCL12	ND	ND

a Molecular weight of the adduct (dye and the phosphopantetheinyl arm of coenzyme A) in Daltons as determined by mass spectrometry following the PPTase reaction, assuming the formation of the appropriate number of disulfide bonds for the given chemokine.

b Theoretical molecular weight of the adduct based on the published structure of the dye.

c CXCL12 mutant with CXCR4 antagonist properties developed in our laboratory (manuscript in preparation).

Abbreviations: PPTase, phosphopantetheinyl transferase. NA, not available. ND, not determined. CPM, 7-diethylamino-3-(4′-maleimidylphenyl)-4-methylcoumarin. NPM, N-(1-pyrenyl)maleimide. GAG, glycosaminoglycan. vMIP-II, viral macrophage inflammatory protein-II.

### Mammalian cell culture and transfection

Jurkat cells (ATCC) and U937 cells (gift of Jeffrey D. Esko, UC San Diego) were maintained in RPMI 1640 media (Invitrogen) supplemented with 10% fetal bovine serum (FBS). HEK293t cells were obtained from ATCC and maintained in Dulbecco's modified Eagle medium (DMEM) with Glutamax (Invitrogen) supplemented with 10% FBS. Transient transfections of these cells with pcDNA3.1-S6-CCR7 and pcDNA3.1-CCR2-YFP were carried out using TransIT-LT1 reagent (Mirus Bio). HEK293s cells used for the scintillation proximity assay were stably transfected with an inducible pACMV-tetO-flag-CXCR4 plasmid and pcDNA6/TR (Invitrogen) according to the manufacturer's protocol and maintained in DMEM/10% FBS with blasticidin and G418. The Chinese hamster ovary cells (CHO-K1) and PGS745 cells (gift of Jeffrey D. Esko, UC San Diego) [23] were maintained in DMEM/F12 nutrient mixture (Gibco) supplemented with 10% FBS. Transient transfections of CHO-K1 cells with pcDNA3.1-Flag-CXCR4, pcDNA3.1-CXCR4-GFP (gift of Adriano Marchese, Loyola University), pmEos2-CCR7 (with CCR7 placed C-terminally to mEos2), and CCR5-mCherry were carried out using the TransIT CHO transfection kit (Mirus Bio).

### Scintillation proximity assay (SPA)

HEK293s cells stably transfected with pACMV-tetO-flag-CXCR4 were treated with doxycycline (2 µg/ml) and sodium butyrate (5 mM) for 24 h to induce CXCR4 expression. The cells were added to a Corning NBS 96-well plate at 50,000 cells per well, along with 0.4 mg of wheat germ agglutinin (WGA) beads (Perkin Elmer), 0.5 µCi ^125^I-CXCL12 (0.2 nM final concentration, Perkin Elmer), and the appropriate amount of unlabeled competitor ligand (WT CXCL12, a P2G-CXCL12 antagonist mutant, or S6-tagged CXCL12) in 20 mM HEPES pH 7.2 containing Hank's Balanced Salt Solution (Gibco) and 0.1% BSA. The cells were incubated at room temperature for 1 h with shaking, and the resulting scintillation was recorded on a MicroBeta TriLux 1450 Scintillation and Luminescence Counter (Perkin Elmer). Triplicate measurements were made for each concentration point, and their average and the SEM (standard error of the mean) were reported in CPM. IC_50_ values were estimated by fitting the data to the one-site model equation: 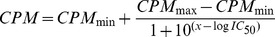
where x is the log of ligand concentration. GraphPad Prism version 5 (GraphPad Software) was used to perform the fit as well as the fits to the calcium mobilization assay described below.

### Calcium mobilization assay

The FLIPR Calcium 4 Assay Kit (Molecular Devices) was used for the calcium mobilization assays. Jurkat cells or U937 cells were centrifuged in a poly-D-lysine-coated 96-well black/clear bottom plate (Becton Dickinson Labware) at 160,000 cells per well and incubated in 200 µl 50 mM HEPES pH 7.4 containing Hank's Balanced Salt Solution (HBBS, Gibco) with the Calcium indicator at 37°C for 90 min as per manufacturer's instructions. Cells were stimulated by the addition of 50 µl HEPES/HBBS containing the appropriate ligand, and their response was recorded for 150 s using a Flex Station 3 plate reader (Molecular Devices). Triplicate measurements were made for each concentration point, and the average of the maximum signal intensity at each concentration and the SEM were reported in Relative Fluorescence Units. EC_50_ values were estimated by fitting the data into the one-site model equation:
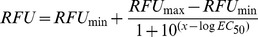
where x is the log of ligand concentration.

### Migration assay

Corning 6.5 mm Transwell plates (24 wells) with 5.0 µm polycarbonate permeable membrane inserts were used for the migration assays. Jurkat cells or U937 cells were resuspended at 2.5×10^6^ cells/ml with RPMI/10% FBS then 100 µl of suspension were added to the inserts and placed over the lower chamber containing 600 µl of the same medium with the appropriate amount of ligand. The number of cells that migrated to the lower chamber after 2 h of incubation at 37°C was recorded by Guava EasyCyte 8HT flow cytometer (EMD Millipore). Triplicate measurements were made for each concentration point, and their average and SEM are reported as the % of the total input.

### Flow cytometry

Surface expression of Flag-CXCR4 in CHO-K1 cells was detected using PE-conjugated rat anti-human CD184 (CXCR4) and the PE-conjugated rat IgG2a κ monoclonal isotype control antibodies (BD Biosciences). Cells were lifted from tissue culture dishes using Cellstripper cell dissociation solution (Cellgro), washed with ice cold PBS+0.1% BSA (FACS buffer), and stained in FACS buffer supplemented with 4 µg/mL of either anti-human CD184 or isotype control for 45 min in the dark on ice. In the case of cell staining with fluorescent CXCL12 derivatives, the same procedure was used, except that the staining FACS buffer was supplemented with 100 nM of an antagonist variant of CXCL12-Alexa647 (LGG-CXCL12, manuscript in preparation); the antagonist was used to ensure that the chemokine treatment would not cause receptor internalization. For both antibody and CXCL12-Alexa647 staining, the cells were washed three times with FACS buffer and fixed with 0.8% paraformaldehyde (PFA) before flow cytometric analysis. For AMD3100 treatment, the FACS buffer was supplemented with 1 µM AMD3100. For heparinase treatment, cells were incubated with 1 µg/ml heparin lyase I, II, and III (Sigma-Aldrich) for 4 h at 37°C and washed with FACS buffer immediately prior to staining with LGG-CXCL12-Alexa647. For heparin washed cells, 100 µg/ml heparin was added to the FACS buffer for the three final washes. The flow cytometry was carried out using a Guava EasyCyte 8HT flow cytometer (EMD Millipore), and the data was analyzed using FlowJo version 7.5.5 (Tree Star, Inc.) and GraphPad Prism version 5 (GraphPad Software). Results were analyzed for significant differences using two-tailed t-tests. The resultant P-values are indicated in the figure legend as follows: n.s., P>0.05; *, P<0.05; **, P<0.01; ***, P<0.005.

### Fluorescence Microscopy

For detection of CXCR4∶CXCL12 localization, 100,000 CHO-K1 cells were seeded overnight onto a 18 mm microscope cover glass pre-coated with human plasma fibronectin (Millipore) in 1 ml 1∶1 DMEM/F12 nutrient mixture (Gibco) with 10% FBS on a 12 well culture plate (Corning) and transfected with pcDNA3.1-CXCR4-GFP the following day. A day after the transfection, cells were stained in 500 µl serum-free media supplemented with 100 nM CXCL12-CPM for 30 min on ice, washed with PBS/0.5% BSA, and fixed with 4% PFA in PBS at room temperature. Cells for the internalization assays were stained and washed as above, incubated in 1 ml serum-free media at 37°C for 30 min, washed with PBS/0.5% BSA and fixed with 4% PFA in PBS. The cover glasses were mounted onto microscope slides with Fluorosave Reagent (Calbiochem).

For detection of CCL2∶CCR2 localization, HEK293t cells were transfected with pcDNA3.1-CCR2-YFP and stained as above, using 100 nM CCL2-Alexa647, with the exception that DMEM was used in the place of DMEM/F12.

For the direct detection of CCL21-Alexa647∶CCR7/GAG interaction, CHO-K1 and PGS745 cells were transiently transfected with pcDNA3.1-CCR7-mEos2. Cells were lifted with 10 mM EDTA in PBS and stained in suspension for 30 min on ice in PBS/0.5% BSA containing CCL21-Alexa647 at concentrations indicated in the main text and figure legends. Stained cells were washed in PBS/0.5% BSA on ice and fixed in 4% PFA at room temperature. Fixed cells were washed in water and resuspended in 70% ethanol and air-dried on a glass cover slip before being mounted onto a slide with Fluorosave Reagent. The CHO-K1 and PGS745 cells expressing CXCR4-GFP were stained with CXCL12-Alexa647 but otherwise treated as above.

These images were collected using an Olympus IX81 DSU spinning disk confocal microscope configured with a PlanApo 60× oil objective and Hamamatsu ORCA-ER digital camera (Hamamatsu, Hamamatsu, Japan). For adherent cell staining, fluorescent images of XY-sections at 0.28 µm were collected sequentially using SlideBook 4.2 software (Intelligent Imaging Innovations, Denver, CO). XY-sections of 1.0 µm were collected for in-suspension staining. The final composite images were created using Photoshop CS4 and Illustrator CS4 (Adobe, San Jose, CA).

For immunofluorescence detection of CCL21, HEK293t cells were transfected with CCR7, cultured, and a subset treated with heparinase (2.5 mU/ml heparin lyases I, II, and III) for 30 min at 37°C. Cells were then harvested using 10 mM EDTA in PBS followed by cytospin onto glass slides (1×10^5^ cells/spot). Samples were then incubated with either 10 nM Alexa647-labeled or WT recombinant human CCL21 (R&D), washed twice with cold PBS, fixed in 4% PFA, blocked with PBS/1%BSA at room temperature for 1 h, and stained with anti-CCL21 (R&D, 1∶100) at 4°C overnight. In some cases, cells were treated with a CCR7 blocking antibody (R&D, 1∶100) during the incubation with the respective recombinant CCL21 species. After several PBS wash steps, the cells were incubated with biotinylated secondary antibody (Vector Labs, 1∶500) at room temperature for 1 h followed by Cy3-conjugated streptavidin (Jackson, 1∶1000). To determine if chemokine was membrane localized, cells were co-stained with wheat germ agglutinin (WGA) conjugated to Alexa 488 (Invitrogen) following the manufacturer's instructions. After washing, the cells were mounted with VectaShield containing DAPI (Vector Labs). Images were recorded with a Nikon Eclipse 80i fluorescence microscope. To assess the extent of interaction, the areas occupied by both CCL21 signal (Red) and DAPI (Blue) were quantified, and the CCL21/DAPI ratio in the untreated sample was defined as 1, and the relative binding in all other samples is expressed as its fraction.

## Results

### Enzymatic Labeling of S6-tagged Chemokine

Previously, we described a method for fluorescent labeling of chemokines by adding an extra cysteine to the C-terminus followed by coupling to maleimide-conjugated fluorophores. The main drawback of this method is low yield because the non-native cysteine causes formation of inappropriate intra- and inter-chemokine disulfides, especially with chemokines that require refolding from inclusion bodies. The goal of this study was to establish a relatively generic and cost-effective alternative method that avoids the need for the introduction of non-native cysteine residues. The site-specific protein-labeling method developed by Yin and coworkers utilizes the phosphopantetheinyl transferase (PPTase) reaction, in which a genetically encoded peptide tag is modified with the phosphopantetheinyl (Ppant) arm of coenzyme A, and, by extension, any molecule that can be coupled to the sulfhydryl group of CoA [19], [20]. The size of the adduct (a 12-residue “S6 tag” and the Ppant arm of CoA) is small relative to chemokines, and it does not rely on an unpaired cysteine. As it can be installed at the C-termini of proteins, we reasoned that it could be a general method for fluorescent labeling of all or most chemokines without significantly affecting function.

**Figure 1:**
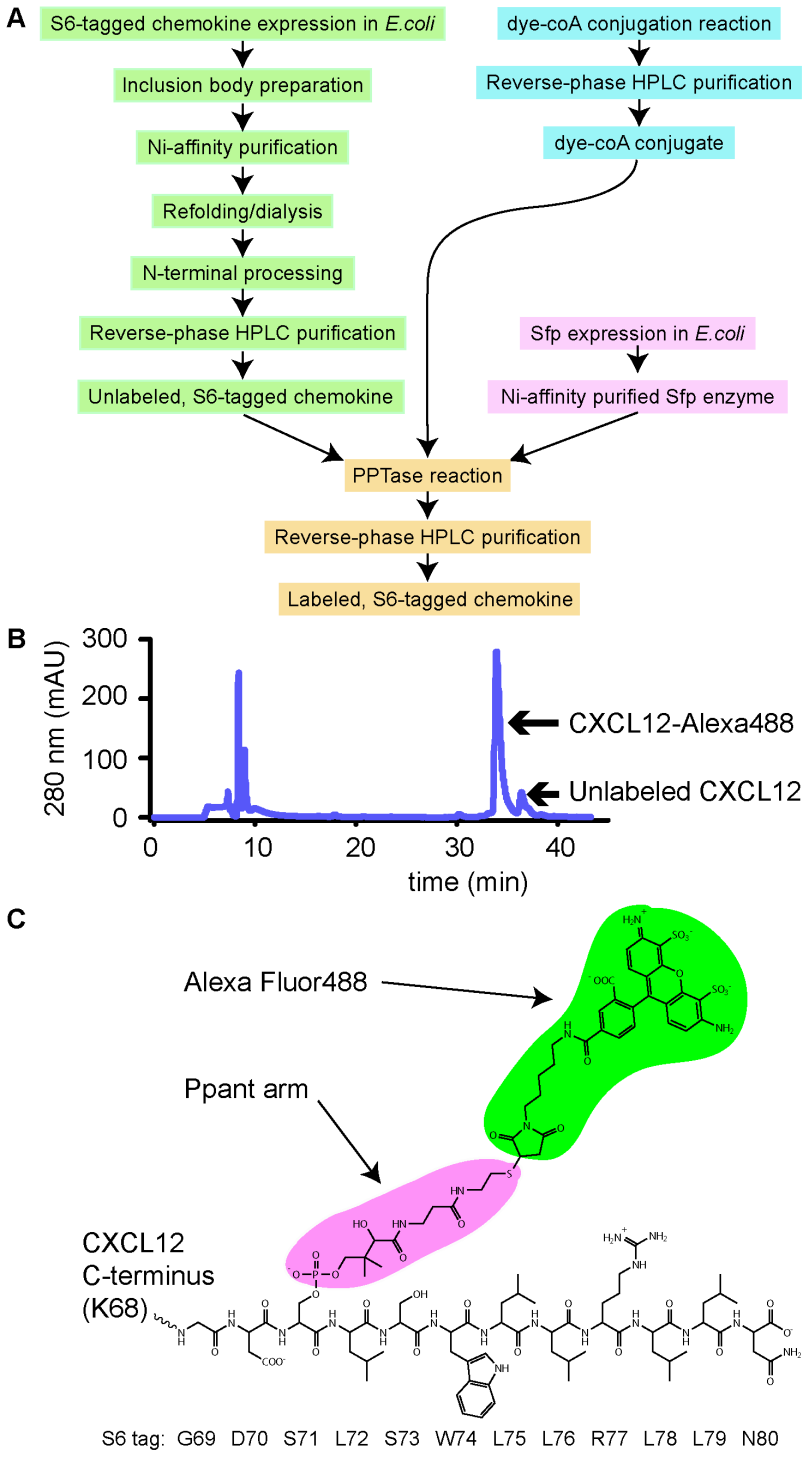
Production of fluorescently labeled chemokines. (A) Overview of the process for fluorescent labeling of chemokine. (B) HPLC trace of CXCL12-Alexa488. (C) A schematic view of the CXCL12-S6 modified by the PPTase reaction in (B). Alexa Fluor488 C_5_ maleimide is colored green. The moiety colored magenta is the phosphopantetheinyl arm of coenzyme A, whose β-phosphate had been attacked by the deprotonated Ser 71 side chain in the S6 tag (sequence GDSLSWLLRLLN; uncolored). The theoretical molecular weight of this adduct is 1038 Da. The S6 tag is fused directly to the C-terminus of CXCL12, comprising the total of 80 residues (9328 Da).

To this end, we generated several expression constructs in which the S6 tag was fused to the C-terminal residue of the chemokine coding sequence (**Table 1**). All of these constructs were expressed insolubly in *E. coli*, purified and labeled according to the scheme shown in **Figure 1a**. The labeling was done according to the published procedure [22], with the exception that the dye-CoA conjugate was used in an equimolar ratio relative to chemokine, as opposed to the 2∶1 ratio specified in the original protocol, in order to maximize the yield with respect to the dye. **Figure 1b** shows the HPLC trace of a typical labeling reaction, in which S6-tagged CXCL12 (CXCL12-S6) was labeled with the CoA-conjugate of Alexa Fluor 488 yielding CXCL12-Alexa488. The trace indicates that there is only a small amount of unlabeled chemokine following the 30 min labeling reaction; thus the reaction can be carried out at a stoichiometric dye-to-protein ratio without sacrificing labeling efficiency. Mass spectrometry analysis of the product showed a molecular weight of 10,367, which is within two daltons of the expected molecular weight. Similar results were obtained with several other chemokines and mutants (**Table 1**), indicating that the PPTase method is highly efficient, specific and that it is potentially applicable to many chemokines. The yield with respect to the input chemokine is ∼40–50%; most losses occur during the labeling reaction where precipitation is often observed, and during the lyophilization step due to sticking to the container. It may be possible to minimize losses at the labeling stage through the use of buffer additives, or alternate tags and PPTase combinations, but we have not yet explored these modifications. Nevertheless, the yield of desired product is quite acceptable in comparison to the traditional cysteine-modification approach.

### CXCL12-S6 is functional

**Figure 2:**
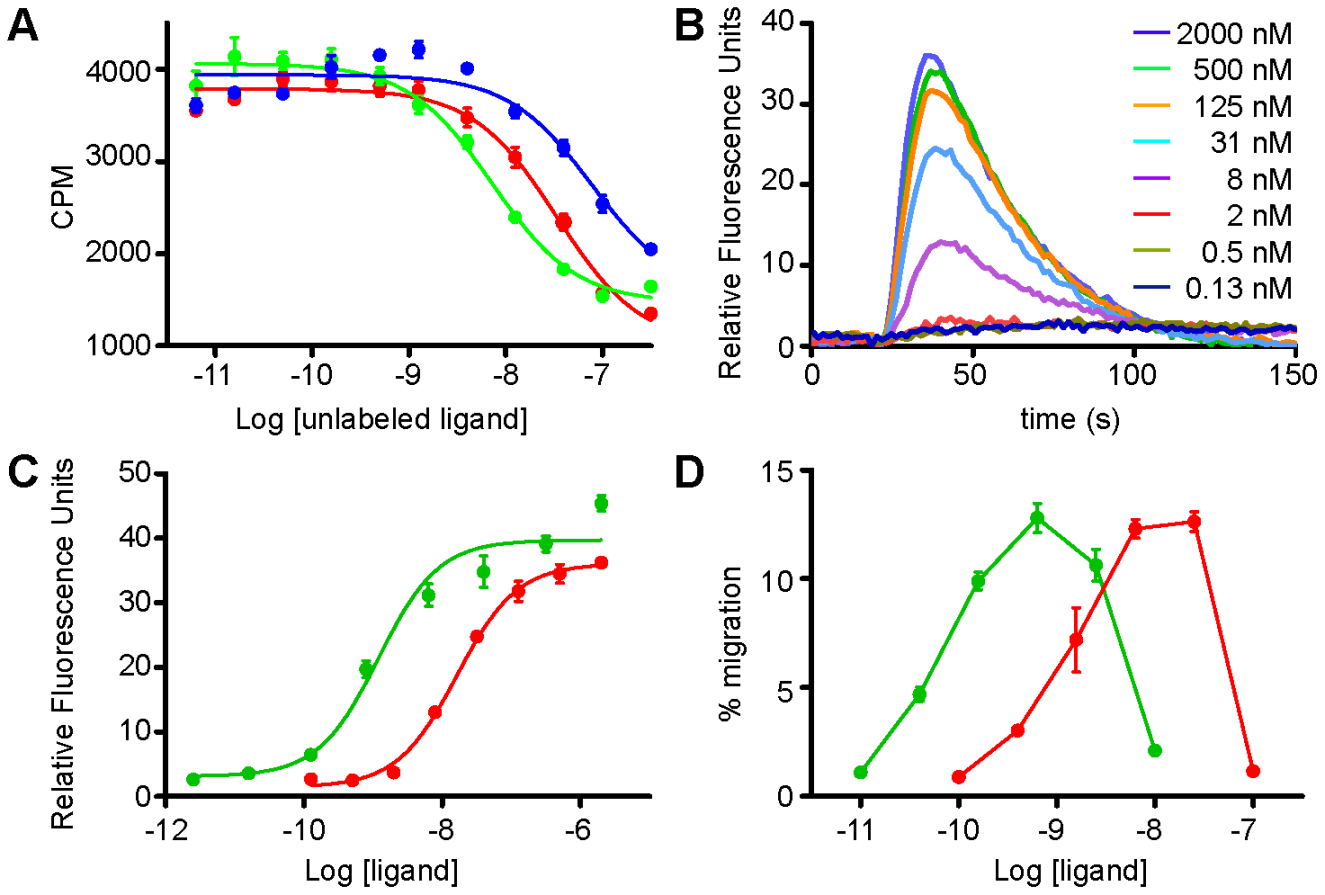
Functional assays of the S6-tagged CXCL12. (A) Scintillation proximity assay of the CXCR4∶CXCL12-S6 interaction. HEK293s cells expressing CXCR4 were incubated with ^125^I-labeled CXCL12, and the amount of cell-bound radioactivity was measured in the presence of WT CXCL12 (green), P2G-CXCL12 (blue) and CXCL12-S6 (red). The IC_50_ was estimated to be 7.2 nM for WT CXCL12, 78 nM for P2G-CXCL12, and 33 nM for CXCL12-S6. (B) Calcium mobilization analysis of the signaling capability of CXCL12-S6. Jurkat cells were stimulated with CXCL12-S6 at the indicated concentrations, and the cytoplasmic calcium release was recorded as a function of time. (C) Calcium mobilization dose-response curve for WT CXCL12 (green) and CXCL12-S6 (red). The maximum signal intensity shown in (B) was plotted as a function of the ligand concentration. The EC_50_ was estimated to be 1.2 nM for WT CXCL12 and 16 nM for CXCL12- S6. . Results of the same experiment with U937 cells are shown in Figure S1a. (D) Migration of Jurkat cells induced by WT CXCL12 (green) and CXCL12-S6 (red). Cells were stimulated with various concentrations of ligands, and the results are expressed as the % of input cells that migrated to the lower chamber of the Transwell plate. Results of the same experiment with U937 cells are shown in Figure S1b.

Having established that the PPTase scheme is an effective alternative to other conventional methods of labeling chemokines with organic fluorophores, we investigated the functionality of the S6-tagged chemokines relative to their WT counterparts. **Figure 2a** shows the result of a scintillation proximity assay (SPA) for determining the binding affinity of WT CXCL12, a P2G antagonist mutant of CXCL12 [24], and CXCL12-S6 for CXCR4 expressed in HEK293 cells. IC_50_ values were 7.2 nM for WT, 78 nM for P2G-CXCL12, and 33 nM for CXCL12-S6. The apparent affinity of WT CXCL12 for CXCR4 has been reported to vary between 1 and 10 nM [25]–[27], and as 9 nM for P2G-CXCL12 [24]. This suggests that the binding affinity determined from our experiment was lower than the actual values by up to ∼9-fold for both WT CXCL12 and P2G-CXCL12. Because CXCL12 requires G protein coupling for high affinity binding [28], we speculate that the reason for the lower affinity observed in this experiment (and related to the range of affinities reported in the literature) is due to overexpression of CXCR4 from the inducible promoter coupled with limiting amounts of heterotrimeric G protein. Regardless of the actual numbers, our results indicate that the affinity of CXCL12-S6 for CXCR4 is lower than WT by ∼5-fold and higher than the P2G-CXCL12 mutant by ∼2-fold. Applying this correction to literature values, we suggest that the affinity of interaction between CXCR4 and CXCL12-S6 is in the 5–10 nanomolar range, which correlates with the results of other functional assays discussed below.

The ability of CXCL12-S6 to activate receptor was then evaluated in a calcium flux assay. In this experiment, Jurkat cells, which express endogenous CXCR4, were stimulated with various amounts of ligand, and the resulting calcium release signal was recorded as a function of time (**Figure 2b**). Non-linear least squares fitting of the dose response curve yielded EC_50_ values of 1.2 nM and 16 nM for the WT CXCL12 and CXCL12-S6, respectively, in good agreement with the estimated binding affinity discussed above (**Figure 2c**).

The ability of CXCL12-S6 to induce cell migration was also examined. In this experiment, Jurkat cells were placed in the top chamber of a bare filter transwell assay setup, and the number of cells that migrated towards chemokine in the lower chamber after 2 h was determined by flow cytometry (**Figure 2d**). Consistent with the binding and calcium flux data, the potency of the CXCL12-S6 is reduced from that of the WT CXCL12 by ∼13-fold, with the maximum migration taking place at ∼12 nM and ∼0.9 nM, respectively. To ensure these observations were not cell-line specific, the calcium flux assay and the migration assay were repeated with U937 cells, which also express endogenous CXCR4, and virtually identical results were obtained for both experiments (**Figure S1**). These results suggest that CXCL12-S6 behaves much like the WT protein, with only slightly reduced functionality due to the reduced binding affinity. As described below, the fluorescent-labeled chemokines are also able to effectively internalize receptor, another indicator of functionality.

### Flow cytometry detection of chemokine interactions with receptors and GAGs

**Figure 3:**
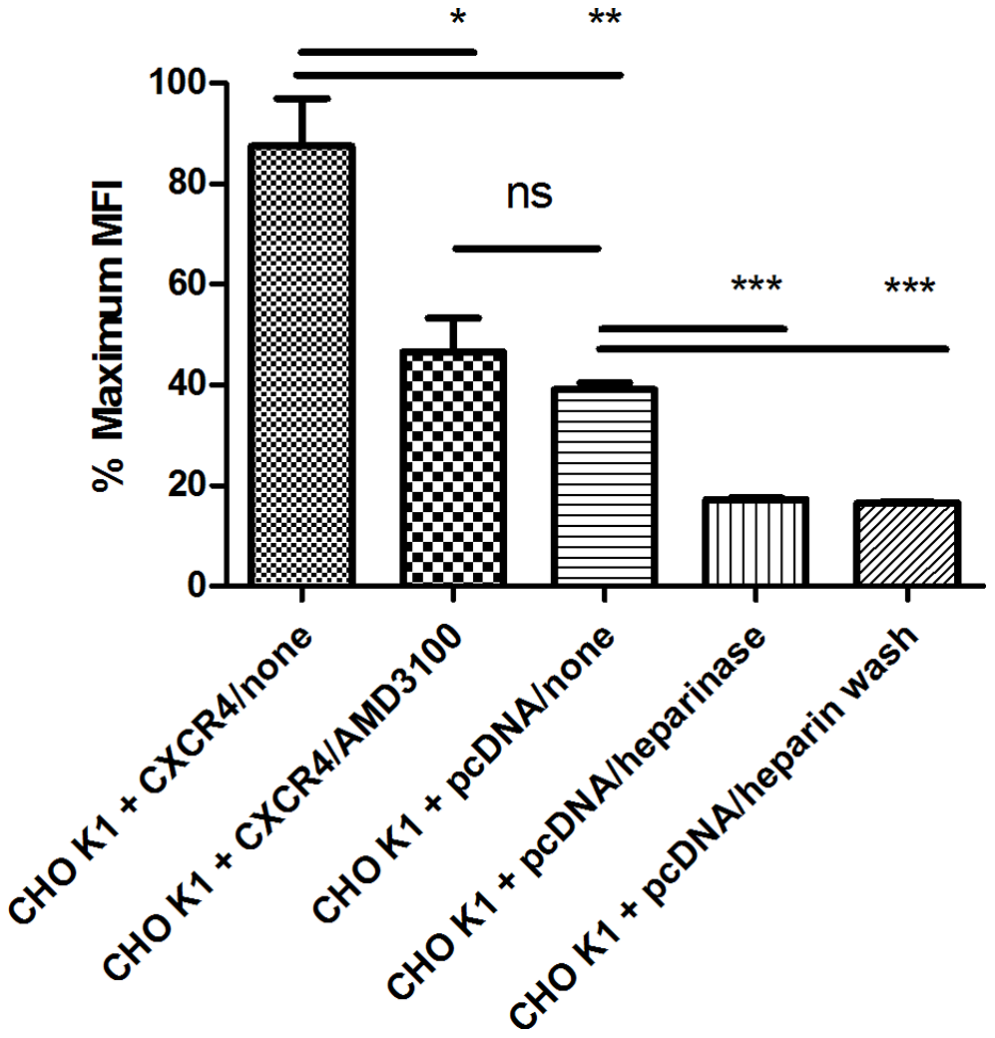
Flow cytometry analysis of the binding of LGG-CXCL12-Alexa647 to CXCR4 and GAGs on CHO-K1 cells. Shown are MFI (median fluorescence intensity) values ± SEM percentage of the maximum, after staining with LGG-CXCL12-Alexa647 in triplicate. Shown from left to right are FLAG-CXCR4-transfected CHO-K1 cells, FLAG-CXCR4-transfeceted CHO-K1 cells stained in the presence of 1 µM AMD3100, pcDNA3.1-transfected CHO-K1 cells, heparinase treated pcDNA3.1-transfected CHO-K1 cells, and pcDNA3.1-transfected CHO-K1 cells washed with heparin after staining. Staining was performed in triplicate and analyzed for significant differences by two-tailed t-tests. The resultant P values are: CHO K1+CXCR4/none vs CHO K1+pcDNA/none, 0.0071; CHO K1+CXCR4/none vs CHO K1+CXCR4/AMD3100, 0.0243; CHO K1+CXCR4/AMD3100 vs CHO K1+pcDNA/none, 0.3315; CHO K1+pcDNA/none vs CHO K1+pcDNA/heparinase, 0.0001; CHO K1+pcDNA/none vs CHO K1+pcDNA/heparin wash, 0.0001. The resultant P-values are indicated within the figure as follows: n.s., P>0.05; *, P<0.05; **, P<0.01; ***, P<0.005.

In order to further establish CXCR4-specific surface binding by the fluorophore-conjugated CXCL12, we stained CHO-K1 cells with an Alexa-647 labeled antagonist variant of CXCL12 (LGG-CXCL12-Alexa647) and performed flow cytometric analysis. We first confirmed that CHO-K1 cells do not express endogenous CXCR4, whereas CXCR4 was detected by rat anti-human CD184 (CXCR4) (BD Biosciences) on the surface of cells transiently transfected with Flag-CXCR4 (**Figure S2**). As expected, surface staining with LGG-CXCL12-Alexa647 was greatest for CXCR4-transfected cells (**Figure 3**). Addition of the competitive small molecule CXCR4 antagonist AMD3100, partially, but not completely, blocked CXCL12 binding. A complete reduction was not expected since chemokines also bind GAGs. Consistent with this, a further reduction in LGG-CXCL12-Alexa647 surface staining of control pcDNA-transfected cells was observed by either washing with heparin or treating with heparinase I, II, and III. Thus both cell surface CXCR4 and GAGs can be fluorescently labeled on live cells using the CXCL12 analogue.

### Confocal microscopy imaging of chemokine∶receptor localization

**Figure 4:**
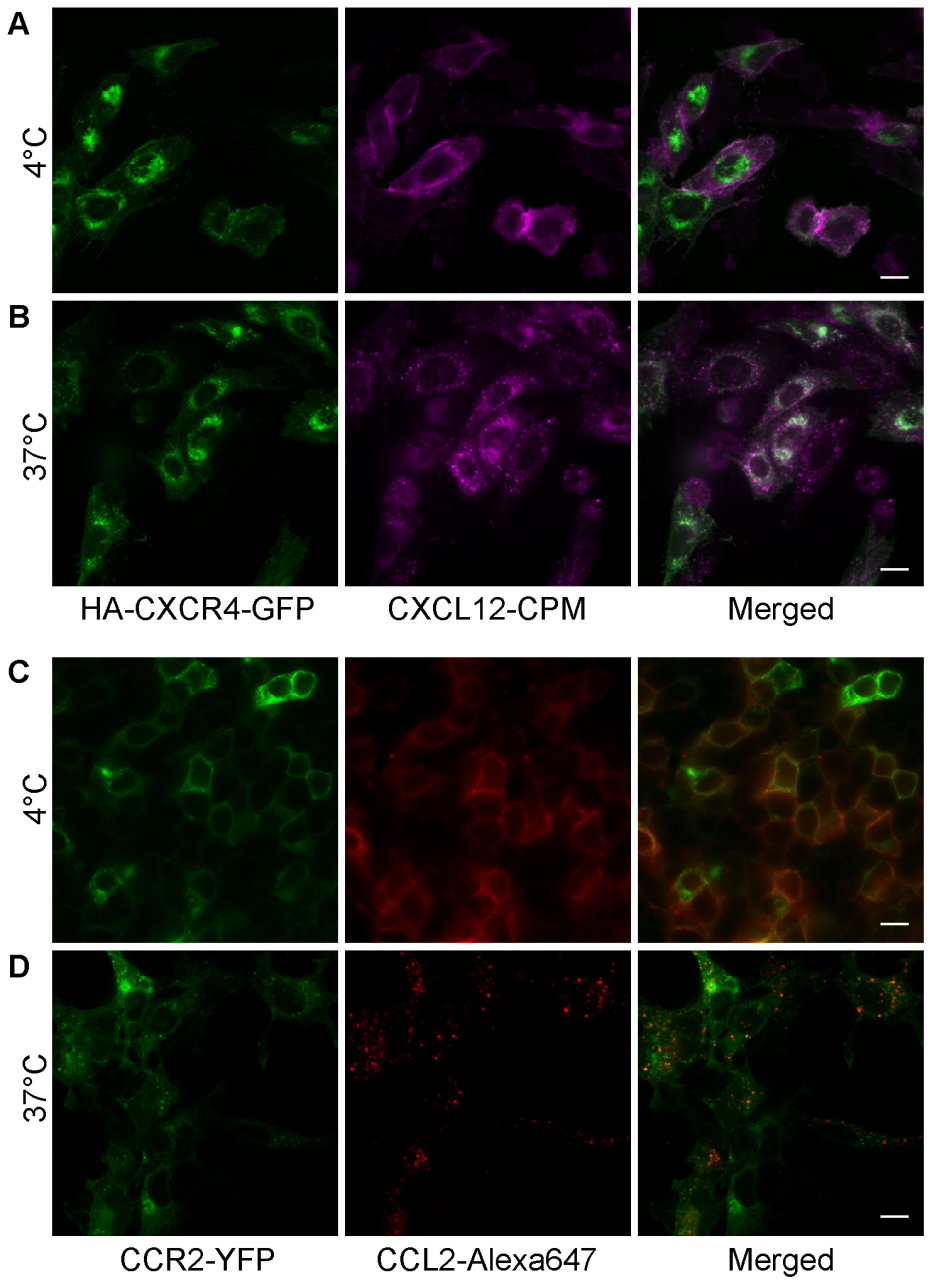
Microscopy imaging of chemokine-receptor interactions and chemokine-mediated receptor internalization. (A) CHO-K1 cells transiently transfected with CXCR4-GFP were stained with 100 nM CXCL12-CPM at 4°C. Left, CXCR4-GFP fluorescence. Middle, CXCL12-CPM. Right, merged image. (B) CHO-K1 cells expressing CXCR4-GFP were incubated at 37°C for 30 min following the surface staining at 4°C with CXCl12-CPM. Left, CXCR4-GFP. Middle, CXCL12-CPM. Right, merged image. (C) Staining of HEK293t cells transiently expressing CCR2-YFP by CCL2-Alexa647 at 4°C. Left, CCR2-YFP fluorescence. Middle, CCL2-S6-Alexa647. Right, merged image. (D) HEK293t cells expressing CCR2-YFP were incubated at 37°C for 30 min following the surface-staining with CCL2-Alexa647 at 4°C. Left, CCR2-YFP. Middle, CCL2-Alexa647. Right, merged image. (Scale bar: 10 µm).

In order to demonstrate the imaging potential of the fluorescently labeled CXCL12 derivatives, we transfected CHO-K1 cells with CXCR4-GFP and stained them with CXCL12-CPM. We note that much of the CXCR4-GFP was detected intracellularly as observed by others for certain cell types [29], [30]. However, a previous study determined that the relative distribution was similar to that of CXCR4 that was not GFP-coupled [30]. As shown in **Figure 4a** (see also **Figure S3**), surface labeling by CXCL12-CPM localizes with CXCR4-GFP fluorescence at the cell surface when the labeling is done at 4°C. Moreover, when the cells stained with CXCL12-CPM were allowed to grow again at 37°C for 30 min, the CPM signal on the cell surface was lost and abundant intracellular puncta appeared, many of which co-localized with the CXCR4-GFP fluorescence, suggesting extensive internalization of CXCL12 and CXCR4 had happened (**Figure 4b**). These data indicate that (i) the fluorescent CXCL12 derivatives can be used to image CXCR4 on cells via microscopy, (ii) that the fluorescent CXCL12 derivatives are functional as they promote receptor internalization, and (iii) that the fluorescent CXCL12 derivatives can be followed through at least the initial endocytosis of the CXCL12-bound receptors.

Essentially the same results were obtained when CCL2-Alexa647 was used to stain HEK293t cells transfected with CCR2-YFP. At 4°C, CCL2-Alexa647 was seen on the surface of cells expressing CCR2 (**Figure 4c**), and virtually no staining was detected on untransfected cells (not shown). After incubation at 37°C, abundant internal puncta were observed, revealing internalization of the labeled chemokine with receptor, and evidence of the functionality of the ligand (**Figure 4d**).

**Figure 5:**
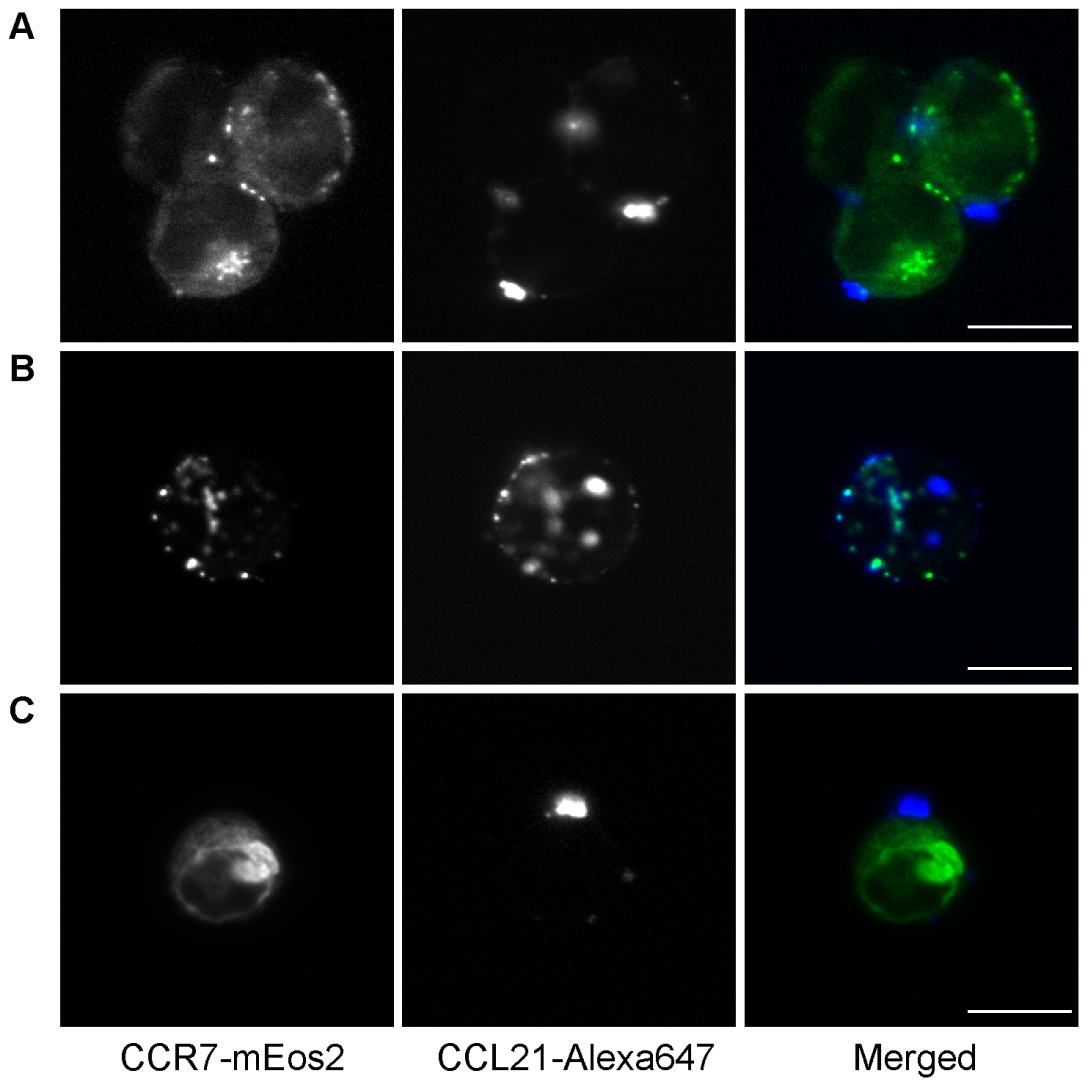
Microscopy imaging of CCL21 on CCR7 expressing cells using CCL21-Alexa647. CHO-K1 cells expressing CCR7-mEos2 were stained in suspension for 30 min on ice with (A) 100 nM, (B) 50 nM, and (C) 25 nM CCL21-Alexa647. Left, CCR7-mEos2 fluorescence. Middle, CCL21-Alexa647. Right, merged image. (A) and (C) show that the puncta are clearly on the outside of the cell. (B) shows that occasionally multiple puncta are observed. (Scale bar: 10 µm).

**Figure 6:**
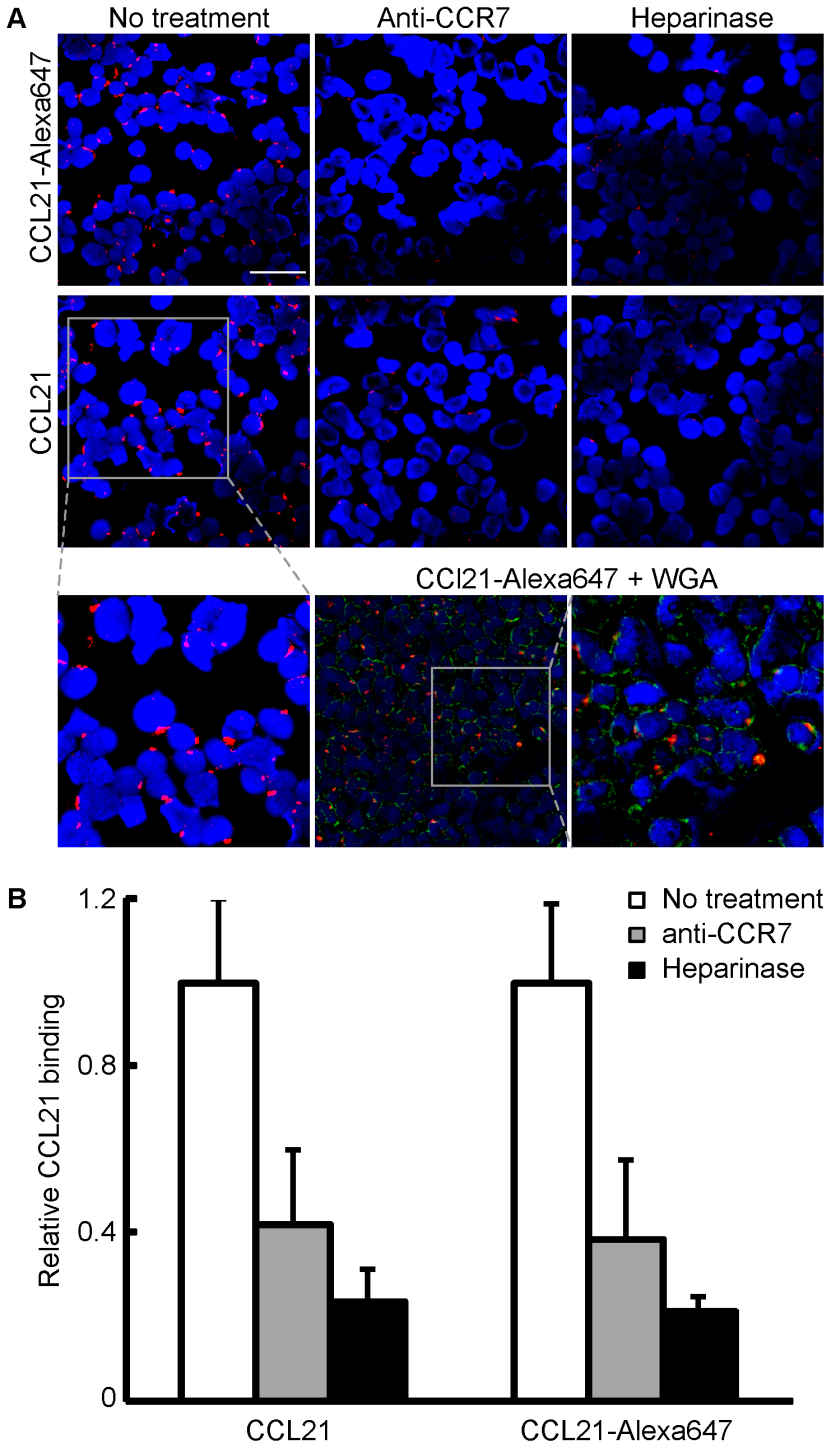
Microscopy imaging of CCL21 on CCR7 expressing cells by immunofluorescence. (A) Binding patterns for both 647-labeled and unlabeled CCL21 (10 nM) show a punctate binding pattern on the surface of HEK293t cells transfected with CCR7. Labeled or unlabeled recombinant human CCL21 were incubated with HEK293t cells, and binding was detected by immunofluorescence staining. Red signal: CCL21; blue signal: DAPI nuclear stain; green signal: membrane staining by wheat germ agglutinin (WGA) conjugated to Alexa-fluor 488(Scale bar: 50 µm). (B) To assess the extent of interaction, the areas occupied by both CCL21 signal (Red) and DAPI (Blue) were quantified, and the CCL21/DAPI ratio in the untreated sample was defined as 1, and the relative binding in all other samples is expressed as its fraction.

CCL21 was also labeled with Alexa647 using the PPTase strategy and used to detect CCR7 on the surface of CHO-K1 cells. In our initial attempts, however, we encountered background staining on the cover slip, on which the transfected cells had been seeded, significantly higher than that observed with CXCL12 or CCL2, and imaging in the adherent settings used in Figure 4 was not suitable for this system. Cells in this experiment were harvested first with EDTA/PBS and then either stained and fixed in suspension before being mounted on the slides (for the direct detection of CCL21-Alexa647) or deposited onto a slide by cytospin prior to staining (for the immunofluorescence detection). As shown in **Figure 5**, the staining of CHO-K1 cells expressing CCR7-mEos2 by CCL21-Alexa647 resulted in an unusual pattern of large puncta on the cell surface. Moreover, cells bearing these large structures generally had only one or two of them. To make sure the results were not an artifact of the CCL21-Alexa647, an alternative secondary immunofluorescence approach was used to visualize the CCL21 localization. In this case CCR7 transfected HEK293t cells were incubated with either WT CCL21 or CCL21-Alexa647 and subsequently treated with the immunofluorescence reagents (see Materials and Methods), and in both cases, the large puncta were again observed (**Figure 6**), indicating that both WT CCL21 and the CCL21-Alexa647 form these structures and that the localization pattern seen in **Figure 5** is not unique to CCL21-Alexa647. Binding sites were on the plasma membrane, as evidenced by co-localization with the membrane marker wheat germ agglutinin (WGA) lectin. Upon treatment with CCR7-blocking antibody or heparinase, the binding of both labeled and WT CCL21 was markedly reduced (**Figure 6a and b**), suggesting that both GAG and CCR7 are required for the formation of these unusual structures and that the fluorescent analogue also retained this aspect.

**Figure 7:**
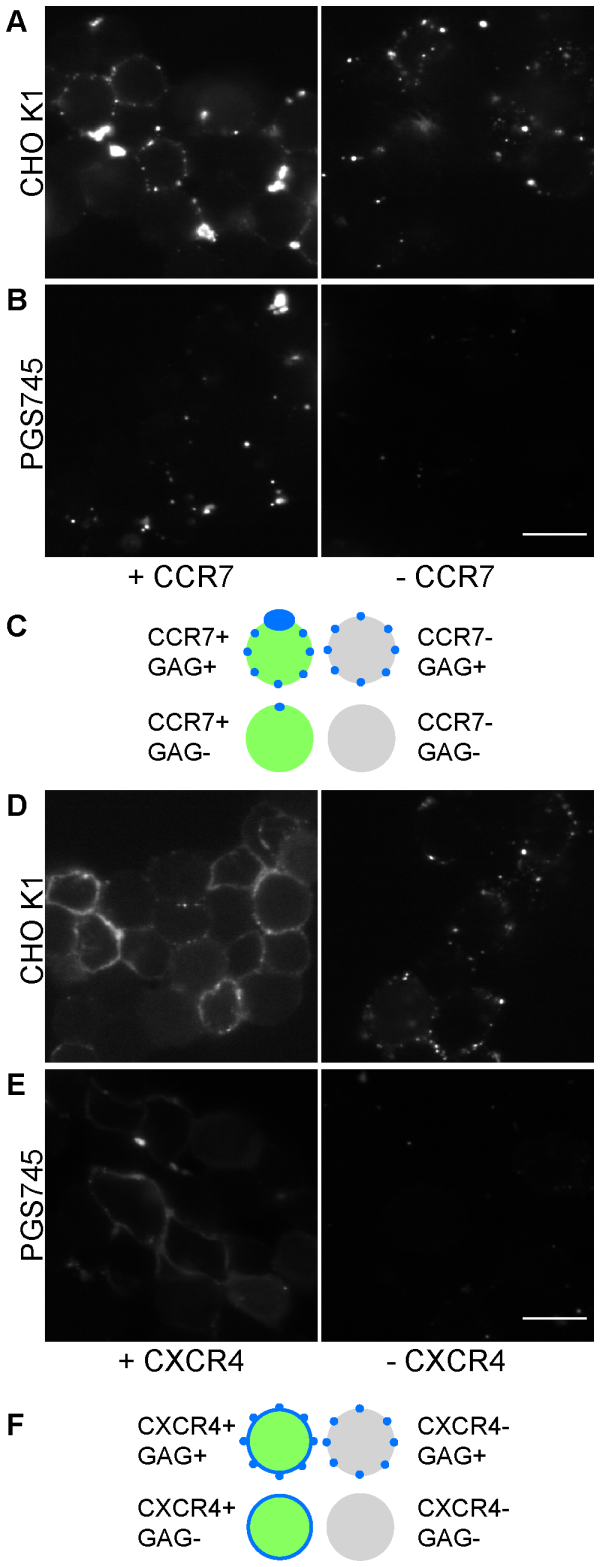
Comparison of CCL21-Alexa647 labeling of CCR7 transfected CHO cells to CXCL12-Alexa647 labeling of CXCR4 transfected CHO cells. (A) CHO K1 cells and (B) the GAG-deficient mutant PGS745 cells were stained with 100 nM CCL21-Alexa647, with (left panel) or without (right panel) the transfected CCR7-mEos2 (Scale bar: 10 µm). Only the chemokine fluorescence is shown for clarity. (C) A cartoon representation of the CCL21 staining patterns observed in (A) and (B) is shown in blue color. Cells expressing CCR7-mEos2 are shown in green. Untransfected cells are colored gray. (D) CHO K1 cells and (E) PGS745 cells were stained with 100 nM CXCL12-Alexa647, with (left panel) or without (right panel) the transfected CXCR4-GFP (Scale bar: 10 µm). Only the chemokine fluorescence is shown for clarity. (F) A cartoon representation of the CXCL12 staining patterns observed in (D) and (E) is shown in blue color. Cells expressing CXCR4-GFP are shown in green. Untransfected cells are colored gray.

To determine if these large CCL21 clusters are chemokine-specific, CXCL12-Alexa647 and CCL21-Alexa647 bound to CHO-K1 cells transfected with their respective receptors were compared using the in-suspension method of staining (**Figure 7**). CXCL12-Alexa647 imaged on CHO-K1 cells transfected with CXCR4-GFP showed a uniform staining of the cell surface, peppered with occasional small puncta distributed across the cell surface (**Figure 7d**, left panel). These small puncta seem to be staining of GAGs, as they are also seen with the untransfected cells (**Figure 7d**, right). Consistent with this notion, staining of PGS745, mutant CHO cells defective in GAG synthesis initiation [31], transfected with CXCR4 resulted in surface staining with a smoother appearance, devoid of the small-puncta pattern (**Figure 7e**, left), suggesting that the former is the component of staining due to the receptor. CCL21-Alexa647 imaged on CHO-K1 cells expressing CCR7, on the other hand, did not show a similar continuous surface stain but rather the large, asymmetric puncta, along with the small structures similar to those seen with CXCL12-Alexa647 on the CHO-K1 cells (**Figure 7a**, left), while the same experiment with the untransfected cells showed only the latter component (**Figure 7a**, right). This indicates that the formation of the large CCL21 cluster is contingent on the expression of CCR7, even though its co-localization with the receptor is not obvious in **Figure 5** (i.e. it does not colocalize with the highest density/brightest spots of CCR7). It also appears, however, that the receptor alone does not support the growth of the large CCL21 puncta, for staining of the PGS745 cells transfected with CCR7 led to asymmetric puncta of reduced sizes (**Figure 7b**, left), again suggesting the role of proteoglycans in these unusual structures.

## Discussion

The goal of this study was to establish a method for fluorescent labeling of chemokines that is cost-effective and straightforward, does not interfere with function, and is generally applicable to many chemokines. The use of the PPTase reaction as a general scheme for labeling of chemokines has several advantages relative to other methods in that the “S6” tag is a small 12-residue tag, the chemokine-S6 tagged constructs can be expressed in bacteria rather than in more complicated eukaryotic systems, and they can be produced as soluble proteins or as insoluble proteins followed by refolding, both without the complications introduced by extra unpaired cysteines. The method also allows one to choose from a wide array of maleimide-conjugated fluorophores as well as other maleimide chemical conjugates.

In this study, the S6-tag was fused to the C-termini of chemokines so as not to disturb their N-termini, which are known to be crucial for receptor activation. All S6 constructs studied here expressed to a similar extent as the untagged constructs and yielded 5–10 mg of purified material from 1 L LB cultures. Thus we have no reason to think this will not be the case for other chemokines, provided that reliable refolding conditions could be worked out. The major source of product loss is during the PPTase conjugation reaction and during the lyophilization step, and while the yields are entirely acceptable from our perspective, there is room for optimization to minimize losses by altering buffer conditions and construct design. We have also observed the loss of labeled products within 2–3 h when reconstituted in water, PBS, or TBS at or below 1 µM. This problem can be alleviated by reconstituting the labeled proteins at 5 µM or above. Other additives such as DMSO, may also resolve this issue if lower stock concentrations are desired.

The results of the functional studies indicate that the S6-tagged CXCL12 retains the activity of the WT protein, albeit with a light reduction in potency. Based on receptor binding competition assays, we estimate the dissociation constant for the interaction between CXCL12-S6 and CXCR4 to be within the 5–10 nM range, which represents a 5–10-fold reduction in affinity. Results of calcium mobilization and migration assays for CXCL12-S6 also demonstrated a slight reduction in potency, which mirrors the reduced affinity. Despite these minor perturbations to chemokine activity, the results show that these labeled chemokines are useful for probing their interactions with receptors and GAGs. We observed that the surface staining of CHO-K1 cells by CXCL12-Alexa647 was strongly dependent on the expression of CXCR4, and the interaction was reduced in response to the treatment with AMD3100, a small molecule inhibitor of CXCR4. Further reduction in binding was observed when the cells were enzymatically treated to digest the GAGs displayed on the cell surface or when the staining was done in the presence of heparin as a competitor. We also found that the receptor-specific binding of the CXCL12-CPM could be visualized by microscopy and that the co-localization of chemokine and receptor could be monitored through the early stages of internalization. Identical results were obtained when HEK293t cells expressing CCR2 were probed with fluorescently labeled CCL2. These findings demonstrate that it is possible to use these reagents to monitor the interactions of chemokines with both receptors and GAGs both in static and dynamic processes such as internalization.

In the case of CCL21, we noticed the presence of unusually large and discrete puncta of CCL21 on CCR7 expressing cells. This cell-staining pattern was observed with direct detection of CCL21-Alexa647 and with secondary-immunofluorescence staining methods applied to both WT CCL21 and CCL21-Alexa647, implying that it is a consequence of CCL21 and not of the fluorescent tag. Direct comparison of CXCL12-Alexa647 with CCL21-Alexa647 also showed that the formation of puncta on these cells is specific and unique to CCL21. Furthermore, formation of the puncta was dependent on both CCR7 and proteoglycan, as blocking with anti-CCR7 antibodies, treatment with heparinase, or use of cells deficient in glycosaminoglycan synthesis, all caused a significant reduction in the presence of these structures. In this regard, it is noteworthy that prior studies characterized CCR7 and heparan sulfate as co-receptors for CCL21 accumulation on hLECs and important for recruitment of CCR7-expressing tumor cells [32].

Interestingly, punctate or large depositions of CCL21 have been noted in other studies aimed at examining the localization of CCL21 in and around human lymphatic endothelial cells (hLECs). Some studies have reported intracellular depots of CCL21 within LECs from *in vivo* imaging of tissue explants of mouse ear sheets and in cultured primary hLECs where it is secreted after TNFα-stimulation [33]. Discrete puncta of CCL21 have been also observed extracellular to initial lymphatic endothelial cells bound to collagen-IV, where the localization was suggested to promote the docking of dendritic cells to the LECs prior to transmigration [34]. Our studies with HEK293 and CHO-K1 cells, and prior studies with primary hLECs [32], suggest that CCR7 and proteoglycans might also cluster CCL21 on cell surfaces, which may contribute to formation of CCL21 gradients.

In summary, we report that the PPTase reaction can be successfully applied to fluorescent labeling of chemokines. They show use in fluorescence imaging by microscopy and flow cytometry. Indeed, a recent study suggests that using fluorescent chemokines may be preferred over fluorescent anti-chemokine receptor antibodies [35]. Microscopy imaging of fluorescently labeled CCL2 and CXCL12 showed the expected patterns of cell surface labeling of receptor-transfected cells. However, CCL21 showed unusual but not unprecedented punctate structures. Thus it seems that the PPTase method holds promise as a general solution for generating fluorescent chemokines to gain information regarding the spatial and temporal behavior of chemokines when the use of exogenously added ligand is the method of choice.

## Supporting Information

Figure S1 **Functional assays of the S6-tagged CXCL12.** (A) Dose response curve of calcium mobilization assay on U937 cells for the WT CXCL12 (green) and CXCL12-S6 (red). (B) Migration of U937 cells induced by the WT CXCL12 (green) and CXCL12-S6 (red).
(TIF)

Figure S2 **CXCR4 expression in CHO cells.** CXCR4 is expressed in CHO-K1 cells only after transfection. The CHO-K1 cells, which were transfected with either FLAG-CXCR4 or empty pcDNA3.1 vector for the experiment shown in Figure 3, were stained with PE-conjugated rat anti-human CD184 (CXCR4) and the PE-conjugated rat IgG2a κ monoclonal isotype control antibodies (BD Biosciences). Shown in the figure are the resultant histograms for isotype control (filled grey) staining, as well as for pcDNA3.1 transfected (grey) and FLAG-CXCR4 transfected (black) cells.
(TIF)

Figure S3 **Microscopy imaging of chemokine-receptor interactions and chemokine-mediated receptor internalization.** Magnified views of the fields shown in Figure 4. (A) CHO-K1 cells transiently transfected with CXCR4-GFP and stained with 100 nM CXCL12-CPM at 4°C. (B) CHO-K1 cells expressing CXCR4-GFP were incubated at 37°C for 30 min following the surface-staining with CXCL12-CPM at 4°C. (C) Staining of HEK293t cells transiently expressing CCR2-YFP by CCL2-Alexa647 at 4°C. (D) HEK293t cells expressing CCR2-YFP were incubated at 37°C for 30 min following the surface-staining with CCL2-Alexa647 at 4°C. (Scale bar: 10 µm).
(TIF)
