# Supplementary figures and images for: A General Method for Site Specific Fluorescent Labeling of Recombinant Chemokines
Source: PLoS One. 2014 Jan 28;9(1):e81454. doi: 10.1371/journal.pone.0081454 (PMC3904831; doi:10.1371/journal.pone.0081454)

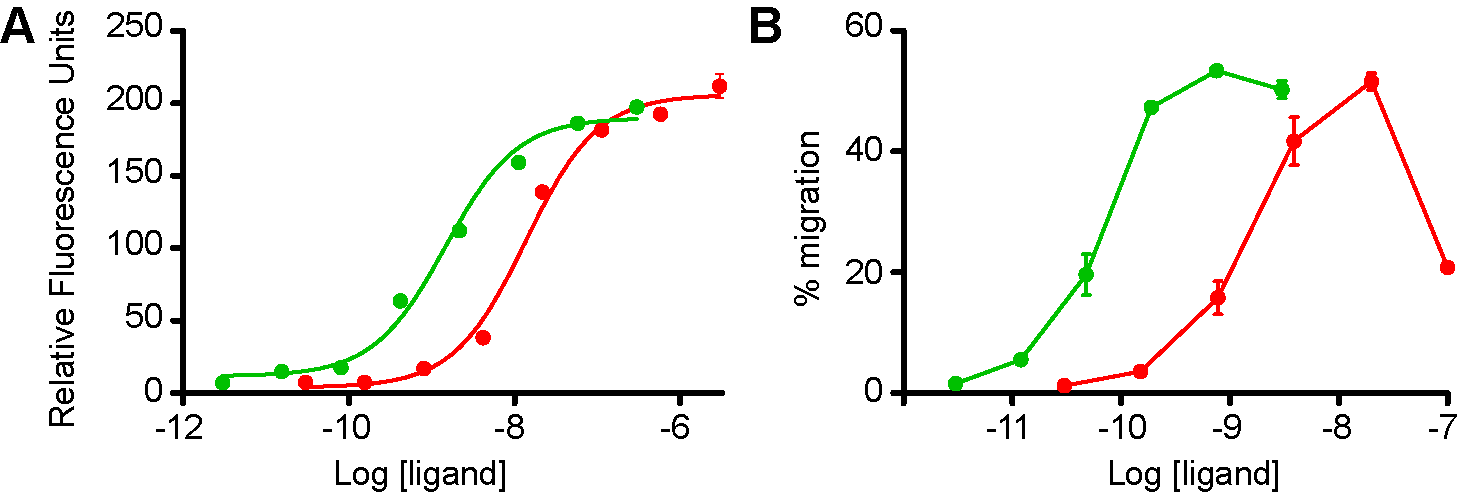

Supplement: Figure S1 — Functional assays of the S6-tagged CXCL12. (A) Dose response curve of calcium mobilization assay on U937 cells for the WT CXCL12 (green) and CXCL12-S6 (red). (B) Migration of U937 cells induced by the WT CXCL12 (green) and CXCL12-S6 (red). (TIF) [file pone.0081454.s001.tif]

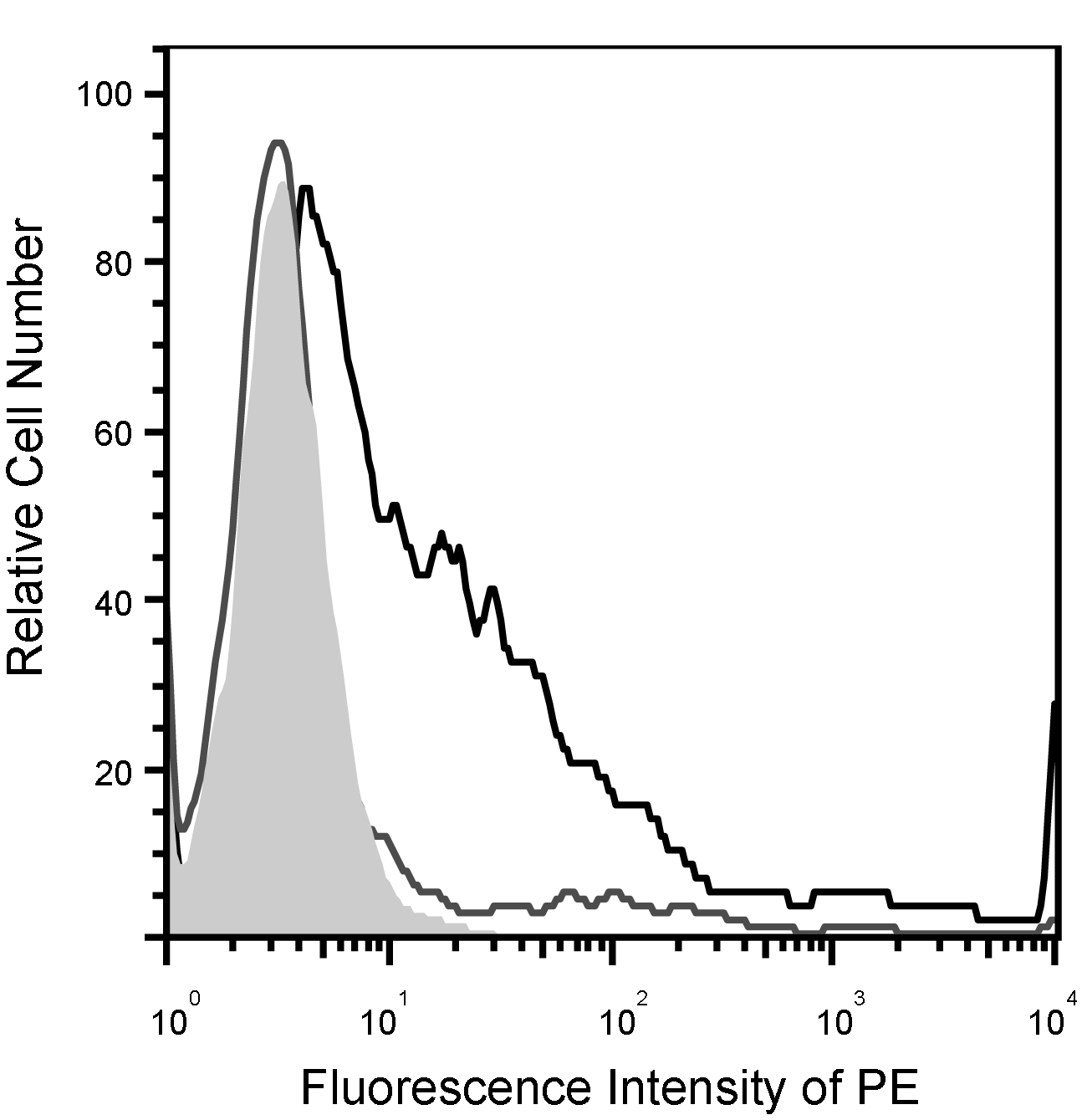

Supplement: Figure S2 — CXCR4 expression in CHO cells. CXCR4 is expressed in CHO-K1 cells only after transfection. The CHO-K1 cells, which were transfected with either FLAG-CXCR4 or empty pcDNA3.1 vector for the experiment shown in Figure 3, were stained with PE-conjugated rat anti-human CD184 (CXCR4) and the PE-conjugated rat IgG2a κ monoclonal isotype control antibodies (BD Biosciences). Shown in the figure are the resultant histograms for isotype control (filled grey) staining, as well as for pcDNA3.1 transfected (grey) and FLAG-CXCR4 transfected (black) cells. (TIF) [file pone.0081454.s002.tif]

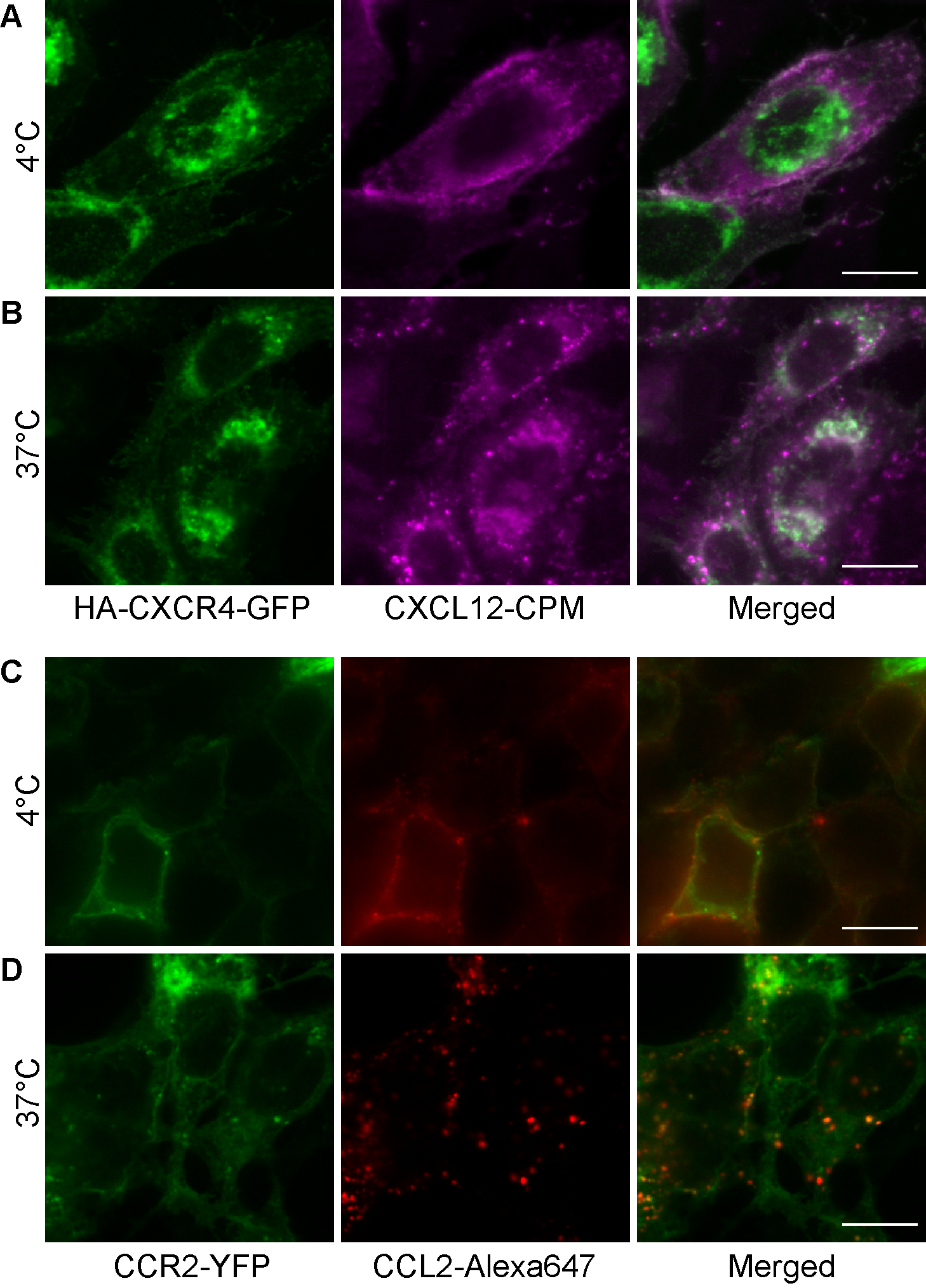

Supplement: Figure S3 — Microscopy imaging of chemokine-receptor interactions and chemokine-mediated receptor internalization. Magnified views of the fields shown in Figure 4. (A) CHO-K1 cells transiently transfected with CXCR4-GFP and stained with 100 nM CXCL12-CPM at 4°C. (B) CHO-K1 cells expressing CXCR4-GFP were incubated at 37°C for 30 min following the surface-staining with CXCL12-CPM at 4°C. (C) Staining of HEK293t cells transiently expressing CCR2-YFP by CCL2-Alexa647 at 4°C. (D) HEK293t cells expressing CCR2-YFP were incubated at 37°C for 30 min following the surface-staining with CCL2-Alexa647 at 4°C. (Scale bar: 10 µm). (TIF) [file pone.0081454.s003.tif]
